# New Diphtheria Outbreak in Europe: A Fulminant and Fatal Case of Respiratory Diphtheria in a 16-Year-Old Patient From Pakistan

**DOI:** 10.7759/cureus.84543

**Published:** 2025-05-21

**Authors:** Marie Berthet, Antoine Costenoble

**Affiliations:** 1 Department of Pediatrics, Cliniques universitaires Saint-Luc, Brussels, BEL

**Keywords:** belgium, corynebacterium diphtheriae, diphtheria antitoxin, migration, pakistan, respiratory diphtheria, vaccination

## Abstract

Diphtheria is primarily a childhood disease that has become rare in Europe. It is caused by *Corynebacterium* bacteria and typically presents in two main clinical forms: respiratory and cutaneous. With timely treatment using antibiotics and anti-diphtheria serum, serious complications and deaths remain uncommon. In this case, a 16-year-old girl from Pakistan was admitted to the ED with pharyngitis but without fever or difficulty breathing. She had no significant medical history, and her vaccination status was incomplete or unknown. Initial treatment with antibiotics and anti-inflammatory medication was started. Twenty-four hours later, she was readmitted due to compensated respiratory failure caused by upper airway obstruction from severe tonsillar hypertrophy. A tonsillectomy and adenoidectomy were performed. However, following surgery, her respiratory condition worsened rapidly, progressing to acute respiratory distress syndrome that required invasive mechanical ventilation. Despite treatment, her condition deteriorated further, necessitating veno-venous extracorporeal membrane oxygenation. The patient developed multi-organ failure that was unresponsive to treatment and died on the sixth day of hospitalization. Autopsy revealed pseudomembranes in the proximal airways, and *Corynebacterium diphtheriae* was identified as the causative pathogen. This case underscores the critical importance of early recognition and appropriate management of diphtheria, a disease now rare in Europe. Prompt administration of antibiotics and anti-diphtheria serum is vital to reduce the risk of severe complications and mortality. Furthermore, it highlights the essential role of vaccination in preventing this disease. Finally, it is crucial to consider diphtheria in the differential diagnosis for patients with unknown or incomplete vaccination histories, such as refugees or asylum seekers.

## Introduction

Historically, diphtheria was a leading cause of infant mortality in Belgium. Since 1959, following the widespread implementation of vaccination, the number of cases has dropped significantly [1]. However, the European Centre for Disease Prevention and Control (ECDC) reports a sharp increase in diphtheria cases across Europe since 2022, with a rising incidence particularly among migrants and asylum seekers [2]. The case described below is one of two fatal diphtheria cases reported in Europe in 2023 and presents with an unusual clinical course.

## Case presentation

On June 23, 2023, a 16-year-old girl was admitted to the ED of a regional hospital due to a sore throat without fever or dyspnea. She was originally from Pakistan and had been living in Belgium for four years at a refugee reception center. She had no notable medical history and was in good health before this episode. Her vaccination status was incomplete. A diagnosis of pharyngitis was made based on the presence of edematous and pustular tonsils, and she was prescribed amoxicillin and ibuprofen for home treatment.

Twenty-four hours later, the patient was readmitted to the same hospital’s ED with compensated respiratory failure caused by upper airway obstruction due to significant tonsillar hypertrophy, without abscess, and marked enlargement of the adenoid tissue. Laboratory tests showed no inflammatory syndrome, and the hemogram was normal. Oxygen therapy was initiated, antibiotic treatment was continued with intravenous amoxicillin and clavulanic acid, and anti-inflammatory treatment with intravenous corticosteroids was started. Despite 48 hours of well-managed treatment, hypoxemia persisted. Due to the obstructive nature of the condition, a tonsillectomy and adenoidectomy were performed on the second day of hospitalization. The surgeon described the tonsils as hepatic and bloody and noted large adenoidal polyps. The patient was transferred to the ICU intubated after the procedure.

Ventilation was easily weaned during the night, and extubation was planned for the following day. However, while preparing for extubation, the intensive care physician found numerous blood clots in the patient’s mouth with diffuse bleeding at the surgical site. The patient was therefore taken back to the operating room for clot removal and cauterization of bleeding in the tonsillar beds, upper turbinates, and nasopharynx. She returned to intensive care sedated and intubated.

During the night, the patient’s respiratory status worsened. She rapidly developed acute respiratory distress syndrome (ARDS) with refractory hypoxemia characterized by bilateral infiltrates on chest X-ray and decreased lung compliance. Concurrently, multi-organ failure developed, including oligo-anuric renal failure, hepatic cytolysis, and disseminated intravascular coagulation. Due to severe refractory hypoxemia despite prone ventilation with FiO2 at 100%, veno-venous extracorporeal membrane oxygenation (VV-ECMO) was initiated. VV-ECMO was placed by cardiac surgeons on the fourth day of hospitalization. The procedure was complicated by misplacement of a venous cannula into the right internal jugular vein, causing right pleural perforation and a compressive right hemothorax. The patient experienced asystole due to the hemothorax upon ECMO initiation, requiring ECMO cessation for one minute of external cardiac massage and administration of 1 mg of adrenaline. The situation stabilized after decompression of the hemothorax with a chest drain, massive transfusion of blood products (RBCs, platelets, and plasma), and significant vasopressor support with noradrenaline. Antibiotic therapy was broadened to include ceftriaxone and clarithromycin.

Once oxygenation was restored, the patient was transferred the same day to the pediatric ICU of a tertiary hospital center while on VV-ECMO. The transfer was uneventful. Upon admission, she presented with multi-organ failure, hemorrhagic shock related to ECMO initiation, decompensated respiratory failure with ARDS, anuric renal failure (creatinine: 3.07 mg/dL), lactic acidosis (lactate: 6.6 mmol/L), hepatic cytolysis (glutamic oxaloacetic transaminase: 2,596 IU/L and glutamate pyruvate transaminase: 1,011 IU/L), biological pancreatitis (lipase: 556 IU/L), and disseminated intravascular coagulation. Laboratory results showed a CRP of 37 mg/L, with neutrophilic hyperleukocytosis (WBC 29.9 × 10³/µL, including 25.2 × 10³/µL neutrophils) (Table 1).

**Table 1:** Laboratory results at the time of admission to the ICU ALT, alanine transaminase; AST, aspartate transferase; GOT, glutamic oxaloacetic transaminase; GPT, glutamate pyruvate transaminase

Laboratory values	Result	Reference range
Hemoglobin	9 g/dL	12-16 g/dL
Platelets	81 × 10³/µL	150-400 × 10³/µL
WBC	29.9 × 10³/µL	5.0-20.0 × 10³/µL
Neutrophils	25.2 × 10³/µL	1.0-9.0 × 10³/µL
CRP	37 mg/L	0-5 mg/L
Creatinine	3.07 mg/dL	0.49-0.84 mg/dL
GOT (AST)	2,596 IU/L	2-77 IU/L
GPT (ALT)	1,011 IU/L	0-41 IU/L
Lipase	556 IU/L	13-60 IU/L
Lactate	6.6 mmol/L	<2 mmol/L

The initial bacteriological workup was negative, as were infectious serologies for cytomegalovirus, Epstein-Barr virus, hepatitis A virus, hepatitis B virus, hepatitis C virus, HIV, and syphilis. Hemorrhagic shock was controlled through significant transfusions of blood products upon admission. However, despite relatively rapid control of hemostasis and hemorrhage, circulatory insufficiency persisted, requiring substantial crystalloid administration, increased vasopressor support with noradrenaline, and the addition of terlipressin. In this context, arterial oxygenation remained adequate under VV-ECMO, and cardiac ultrasound showed good biventricular function without signs of myocardial failure. The pulmonary condition worsened, with the development of severe pulmonary edema necessitating high ventilation pressures.

On the fifth day of hospitalization, the patient’s circulatory failure continued to deteriorate despite ongoing fluid administration and initiation of continuous adrenaline infusion. Due to this persistent decline, she was taken to the operating theater for veno-arterial ECMO (VA-ECMO) insertion. Upon opening the chest, her hemodynamic parameters improved, allowing for a reduction in vasopressor support. Given the clinical improvement after sternotomy, the decision was made not to proceed with VA-ECMO at that time.

However, upon returning to intensive care, she developed decompensated circulatory failure marked by hypotension and bradycardia. VA-ECMO was then placed at the bedside. Although her blood pressure was quickly stabilized with ECMO, peripheral circulation remained critically compromised. Abdominal compartment syndrome developed and persisted despite drainage of a large volume of ascitic fluid. In the following minutes, the patient rapidly developed progressive bradycardia, followed by treatment-refractory asystole. Given the irreversible nature of her condition, ECMO was discontinued, and the patient was pronounced dead in the presence of her family.

Autopsy revealed pseudomembranes in the proximal airways, diffuse pulmonary congestion, and large pleural and peritoneal effusions. Due to the presence of pseudomembranes, microbiological testing for *Corynebacterium diphtheriae* was performed and returned positive several days later.

Once the pathogen was identified, the case was reported to Belgian health authorities, who conducted contact tracing. Those exposed were treated with azithromycin and vaccinated if their immunization was incomplete.

A few days after the patient’s death, her sister presented with similar symptoms but showed rapid improvement following early administration of anti-diphtheria serum.

## Discussion

Diphtheria is caused by *Corynebacterium* bacteria, most commonly *C. diphtheria*, which has a human reservoir. Less frequently, *Corynebacterium ulcerans *and *Corynebacterium pseudotuberculosis*, which have animal reservoirs, can also cause diphtheria [1,3]. These bacteria may or may not carry the gene encoding diphtheria toxin (tox) and may or may not produce the toxin itself [1,3]. Diphtheria mainly presents in two clinical forms: respiratory and cutaneous. The most common form, diphtheric angina, presents with dysphagia, fever, and the formation of false membranes on the tonsils. These membranes can be extensive, bleed, and obstruct the larynx, potentially causing death by asphyxia. They may also extend into the trachea and bronchi. The cutaneous form is characterized by false membranes on pre-existing wounds and is often less severe. Toxin-producing (tox+) corynebacteria can disseminate the toxin throughout the body, with primary binding sites including the myocardium and peripheral nervous system, potentially causing myocarditis and peripheral paralysis [1]. Other clinical presentations, such as septicemia, have also been reported [4]. Transmission occurs through airborne respiratory droplets or direct contact with cutaneous lesions.

Diphtheria remains endemic in some Eastern European countries (e.g., Russia) and other regions, including South America (Brazil and Ecuador), East Asia (Thailand, India, Indonesia, and Nepal), and Africa (Algeria, Madagascar, and Zambia) [5]. Historically, diphtheria was a major cause of infant mortality in Belgium. However, since the widespread introduction of vaccination in 1959, the number of cases has declined significantly [1]. Between 1950 and 1959, there were 7,412 cases, including 370 deaths, but this dropped to fewer than 15 cases between 1980 and 2010 [1]. A similar trend is observed across Europe, where the disease is now rare. Between 2016 and 2020, 128 cases were reported in Europe, with the last diphtheria-related death in Belgium occurring in 2016, involving an unvaccinated three-year-old girl [6].

Nevertheless, according to the ECDC, diphtheria cases in Europe increased sharply in 2022, with 359 cases reported, including 31 in Belgium [2,7]. From January to December 12, 2023, 121 cases were reported across Europe (six in Belgium, including our patient), with 99 caused by *C. diphtheriae *and 32 by *C. ulcerans *[7]. In December 2023, of the 131 total cases, 113 were cutaneous, 13 respiratory, 2 both cutaneous and respiratory, and 3 were classified as “other clinical presentations.” Two fatal cases were reported, including our patient, both caused by respiratory *C. diphtheriae *infections [7].

The clinical presentation of our patient was unusual. Death from diphtheria is typically due to asphyxia caused by upper airway obstruction; cases involving systemic infection with multi-organ failure are rare. A similar case was reported in Austria in May 2022, involving multi-organ failure due to a tox+ *C. diphtheriae *infection with initial respiratory symptoms requiring ECMO treatment [8].

The ECDC also reports an increase in diphtheria among migrants and asylum seekers. In 2022, of the *C. diphtheriae *cases with known importation status, 62% were classified as imported (patients outside the country during incubation and with no local transmission), and 3.3% were linked to imported cases [2]. Of the 131 cases reported in 2023, 46 were classified as imported (from Afghanistan (21), Syria (nine), Sudan (two), Croatia (one), Ethiopia (one), Indonesia (one), Iraq (one), the Philippines (one), Slovenia (one), Thailand (one), and unknown origin (seven)), eight were linked to imported cases, 36 were not imported, and 41 had unknown import status [7].

Regarding treatment, first-line therapy involves antibiotics such as penicillin or macrolides for allergic patients [9]. Antibiotics inhibit toxin production and limit disease transmission but do not neutralize circulating toxin, so they cannot reverse established damage. Since the diphtheria toxin causes irreversible damage once it enters cells, timely administration of diphtheria antitoxin is critical to reduce mortality and complications. The antitoxin is ineffective once the toxin binds to target cells and therefore must be given as early as possible, ideally at suspicion of the disease [9]. In our patient’s case, the bacteria were identified only postmortem, so toxin presence is unknown, but the severity suggests toxin production; early antitoxin treatment could have potentially slowed symptom progression.

The ECDC emphasizes that universal immunization is the only effective preventive measure against toxin-mediated diphtheria and that fully vaccinated individuals have a very low risk of developing the disease [7,10]. In 2021, the vaccination coverage for the third dose of diphtheria-tetanus-pertussis vaccine ranged from 85% to 99% across Europe and was 97.5% in Belgium [11]. The patient’s vaccination status is unknown. In Pakistan, her country of origin, the vaccination rate was 85% in 2022 but only 54% in the patient’s first year of life [12]. In 2022, Pakistan reported 351 diphtheria cases [12]. Among European cases in 2022, vaccination status was documented for only 39% of patients [2], with 75% of those either unvaccinated or incompletely vaccinated [2]. Despite the low likelihood of disease in Belgium due to high vaccine coverage, diphtheria should be considered when evaluating patients with typical presentations, especially those living in refugee centers.

## Conclusions

Europe is currently experiencing an increase in diphtheria cases, with a notable rise among migrants and asylum seekers. While complications in the respiratory form of diphtheria, especially with toxin-producing strains, are common, death due to multi-organ failure, as seen in our patient, is rare. Early recognition of clinical signs is essential to ensure prompt and effective treatment. Moreover, rapid administration of anti-diphtheria serum alongside appropriate antibiotic therapy is critical to reducing the risk of complications and mortality. Finally, since vaccination remains the only effective preventive measure, special attention must be given to ensuring full vaccination of refugees and asylum seekers who may not have been immunized in their countries of origin.
